# A case of subconjunctival dirofilariasis in South India

**DOI:** 10.1007/s12348-012-0078-6

**Published:** 2012-05-11

**Authors:** Savita Bhat, Ovi Sofia, M. Raman, Jyotirmay Biswas

**Affiliations:** 1 Giridhar Eye Institute, Kochi, India; 2 Department of Ophthalmology, Saiful Anwar General Hospital, Brawijaya University, Malang, Indonesia; 3 Department of Parasitology, Madras Veterinary College, Chennai, India; 4 Department of Uveitis and Ocular Pathology, Sankara Nethralaya, Chennai, India

**Keywords:** Subconjunctival dirofilariasis, Dirofilaria tenuis

## Abstract

We describe a case of subconjunctival dirofilariasis from South India. The worm was identified by detailed morphologic study.

**Electronic supplementary material:**

The online version of this article (doi:10.1007/s12348-012-0078-6) contains supplementary material, which is available to authorized users.

A 75-year-old male presented with redness and irritation of the left eye. Ophthalmic examination revealed a thin white live worm wriggling around superior conjunctiva. The worm removal was done under local anesthesia and identified as an adult female *Dirofilaria tenuis*. *D. tenuis* rarely causes subconjunctival dirofilariasis in Asia. The parasite was identified by detailed morphologic study in wet preparation. Dirofilaria is identified by filariform; body cuticle is finely striated. Mouth without lips and encircled by six to ten papillae and head papillae are insignificant. Esophagus is relatively short and very distinctly divided into two portions—muscular and ventricular (Figs. 1, 2, and 3). The identification of the parasite can also be done using polymerase chain reaction technique.

**Fig. 1:**
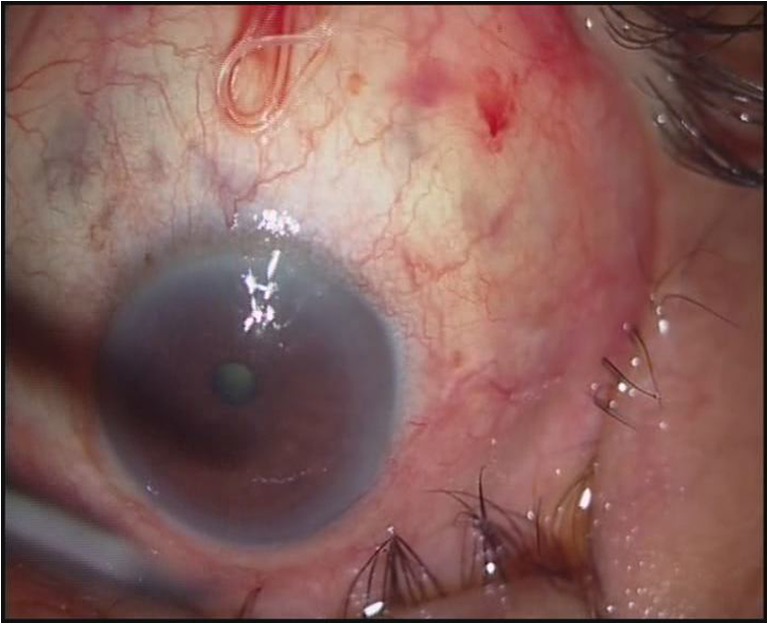
Photograph of left eye showing the live worm in superior conjunctiva

**Fig. 2:**
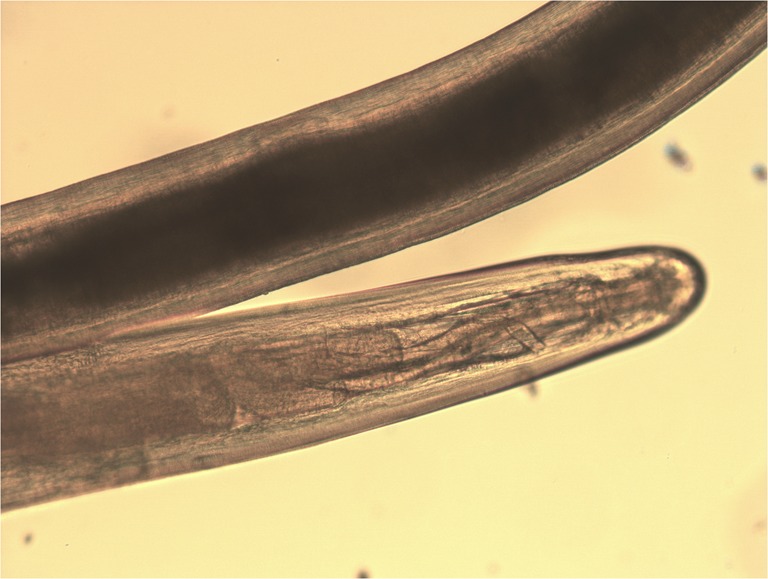
Anterior end of adult female *D. tenuis* (×10)

**Fig. 3:**
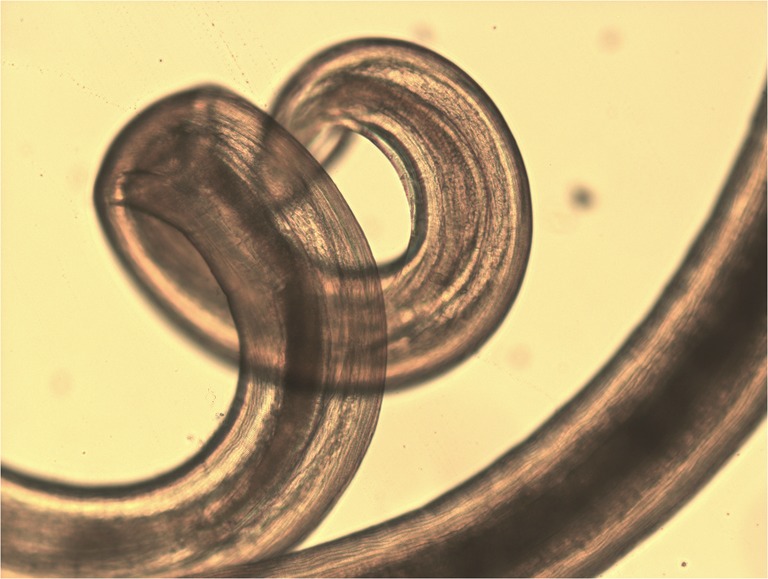
Posterior end of adult female *D. tenuis* (×10). Posterior end is rounded and vulva is little behind the esophagus

## Electronic supplementary material

Below is the link to the electronic supplementary material.

Video 1 Video showing the surgical removal of the worm (MPG 9,945 kb)

